# Evolution and Diversity of the Human Leukocyte Antigen(HLA)

**DOI:** 10.1093/emph/eou033

**Published:** 2015-01-10

**Authors:** Peter V. Markov, Oliver G. Pybus

**Affiliations:** ^1^ Epidemiology of Emerging Infections Unit, Institut Pasteur, Paris, France; ^2^Department of Zoology, University of Oxford, Oxford, UK

## Human leukocyte antigen

Human leukocyte antigen (HLA) Class I and Class II antigens can be genetically extremely diverse, play a central role in immunity and are of primary importance in several branches of medicine. By presenting fragments of pathogens or altered ‘self’ proteins, HLA molecules activate T lymphocytes and are essential in adaptive immune responses against infection or cancer. Clinically, certain HLA types are associated with severity, treatment outcomes or risk of particular infections (e.g. HIV, leprosy, hepatitis B virus) [1]. HLA are the most important histocompatibility antigens to match donors and recipients for successful organ or tissue transplantations. They are also the strongest known predictors of a range of autoimmune disorders including Type I diabetes, coeliac disease, narcolepsy and spondyloarthritis [2]. Increasing evidence also suggests that the combination of HLA types in a couple might affect fertility [3].


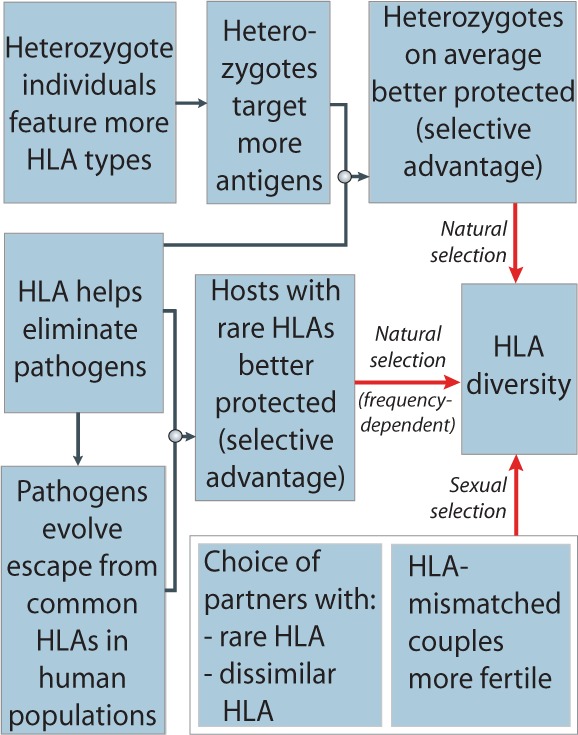


## Evolutionary perspectives

With > 7000 alleles HLA is the most polymorphic region of the human genome [4]. The common view is that this extraordinary diversity is an evolved defence of slowly evolving vertebrates against rapidly evolving microparasites [5].

Each HLA type ‘sees’ a unique set of pathogen targets (epitopes). Individuals carrying different alleles at a given HLA locus (heterozygotes) produce both molecules thereby increasing the number of potentially targeted epitopes and so, on average enjoy broadened immunity and likely stronger protection [5]. Such advantage means that everything else being equal, heterozygosity at HLA loci will be favoured by natural selection. Furthermore, when evolving pathogens infect HLA-heterogeneous populations they are forced to adapt *de novo* each time they transmit. This hinders the adaptation of pathogens to the population of hosts as a whole and also favours in hosts many, rare HLA alleles [5]. HLA diversity is further promoted through reproductive mechanisms. Evidence from studies in animals and humans indicates bias in mate choice towards partners carrying major histocompatibility complex (MHC) or HLA alleles that are rare and/or dissimilar from the individual’s own. Couples more discordant in their HLA alleles were shown to be more fertile [3]. Such behavioural and reproductive phenomena may be selectively favoured if they increase HLA diversity of offspring.

## Future implications

Studies of HIV and Hepatitis C virus suggest that rapidly evolving RNA viruses are able to escape HLA-dependent defences in human populations [6]. HLA is a major determinant of viral persistence and severity while many current vaccine efforts employ HLA-restricted immune responses. Thereby proper understanding of virus-HLA coevolution is instrumental for making informed treatment and vaccine-design decisions for a range of major chronic and acute infections. Given the frequency-dependent protection of HLA, populations where particular HLA types are very common (e.g. A*2301 in West Africa) could potentially be identified as hot spots for severe disease, endemic persistence or pathogen emergence.

## References

[1] Chapman SJ, Hill AV (2012). Human genetic susceptibility to infectious disease. Nat Rev Genet.

[2] Dyer P, McGilvray R, Robertson V (2013). Status report from ‘double agent HLA’: health and disease. Mol Immunol.

[3] Ober C (1999). Studies of HLA, fertility and mate choice in a human isolate. Hum Reprod Update.

[4] Robinson J, Halliwell JA, McWilliam H (2013). The IMGT/HLA database. Nucleic Acids Res.

[5] Sommer S (2005). The importance of immune gene variability (MHC) in evolutionary ecology and conservation. Front Zool.

[6] Kawashima Y, Pfafferott K, Frater J (2009). Adaptation of HIV-1 to human leukocyte antigen class I. Nature.

